# Healthcare utilization and catastrophic health expenditure in rural Tanzania: does voluntary health insurance matter?

**DOI:** 10.1186/s12889-023-16509-7

**Published:** 2023-08-17

**Authors:** Alphoncina Kagaigai, Amani Anaeli, Sverre Grepperud, Amani Thomas Mori

**Affiliations:** 1 Institute of Health and Society, University of Oslo, P.O. Box 0315, Oslo, Norway; 2 School of Public Health and Social Sciences, Muhimbili University of Health and Allied Sciences, P.O. Box 65001, Dar Es Salaam, Tanzania; 3 Department of Global Health and Primary Health Care, University of Bergen, P.O. Box 5007, Bergen, Norway

**Keywords:** Tanzania, Catastrophic health expenditure, Community-based health insurance scheme, Cross-sectional household survey, Out-of-pocket expenditure, Concentration index

## Abstract

**Background:**

Over 150 million people, mostly from low and middle-income countries (LMICs) suffer from catastrophic health expenditure (CHE) every year because of high out-of-pocket (OOP) payments. In Tanzania, OOP payments account for about a quarter of the total health expenditure. This paper compares healthcare utilization and the incidence of CHE among improved Community Health Fund (iCHF) members and non-members in central Tanzania.

**Methods:**

A survey was conducted in 722 households in Bahi and Chamwino districts in Dodoma region. CHE was defined as a household health expenditure exceeding 40% of total non-food expenditure (capacity to pay). Concentration index (CI) and logistic regression were used to assess the socioeconomic inequalities in the distribution of healthcare utilization and the association between CHE and iCHF enrollment status, respectively.

**Results:**

50% of the members and 29% of the non-members utilized outpatient care in the previous month, while 19% (members) and 15% (non-members) utilized inpatient care in the previous twelve months. The degree of inequality for utilization of inpatient care was higher (insured, CI = 0.38; noninsured CI = 0.29) than for outpatient care (insured, CI = 0.09; noninsured CI = 0.16). Overall, 15% of the households experienced CHE, however, when disaggregated by enrollment status, the incidence of CHE was 13% and 15% among members and non-members, respectively. The odds of iCHF-members incurring CHE were 0.4 times less compared to non-members (OR = 0.41, 95%CI: 0.27–0.63). The key determinants of CHE were iCHF enrollment status, health status, socioeconomic status, chronic illness, and the utilization of inpatient and outpatient care.

**Conclusion:**

The utilization of healthcare services was higher while the incidence of CHE was lower among households enrolled in the iCHF insurance scheme relative to those not enrolled. More studies are needed to establish the reasons for the relatively high incidence of CHE among iCHF members and the low degree of healthcare utilization among households with low socioeconomic status.

**Supplementary Information:**

The online version contains supplementary material available at 10.1186/s12889-023-16509-7.

## Background

Globally, the proportion of total health expenditure is less than 10% of the Gross Domestic Product (GDP). Additionally, the proportion of out-of-pocket (OOP) health expenditure has remained above 40% of the total health spending in low and middle-income countries (LMICs) [1]. It is commonly considered that OOP payments that exceed 10% of a household’s income or 40% of a household’s total non-food expenditure often referred to as capacity to pay, represents catastrophic health expenditures (CHE) [1–3]. From 2010 to 2015, the global population that incurred CHE at a 10% income threshold, increased from about 570 million to more than 900 million. When the 25% income threshold was used, the number of people with CHE increased from about 100 million to about 200 million in the same period. Furthermore, about 90 million people (1.2%) were pushed into extreme poverty (spending below $1.90 per person per day) due to OOP health spending in 2015 [4]. The largest number and percentage of the world population impoverished by OOP health spending are from countries in Asia and Africa [4].

The majority of people in some LMICs, particularly low-income earners rely on public health facilities for affordable services [2]. However, public health systems face many challenges including low quality of care, frequent stock-outs of essential medicines, and shortage of healthcare workers [5], hence forcing patients to seek costly services from private health facilities. Unfortunately, health insurance coverage is low in most LMICs, hence most people are unprotected from unexpectedly high healthcare costs [1]. As a result, OOP continues to be the main means of healthcare financing, thus exposing many people to CHE [6–8]. In Tanzania, OOP accounts for about 22% of the total health expenditure, while health insurance schemes (premium payment) account for about 8% [9].

The challenge of raising sufficient funds to finance healthcare is one of the major reasons for LMICs not being able to meet the healthcare needs of their citizens [10, 11]. Community-based health insurance schemes (CBHIs) represent one important strategy for protecting rural and informal sector workers from impoverishing OOP payments [11–13]. According to the WHO, CBHIs are micro health insurance schemes primarily targeted at low-income households. Generally, the pooling of health risks occurs within a community or a group of people that share common characteristics such as geographical location or occupation. The membership premiums are typically flat rates (independent of individual health risks) and the schemes operate on a non-profit basis [14–17]. However, such schemes have not been always successful in providing an adequate level of financial protection [16, 18, 19]. Limited financing sources, the absence of scheme promotion initiatives, and the lack of governmental commitment have contributed to the limited growth of CBHIs, thus delaying the progress toward universal health coverage (UHC) [18].

In Tanzania, the CBHI scheme, commonly referred to as Community Health Fund (CHF), was introduced in 1996 to enhance access to primary healthcare services among rural and informal workers [15]. Despite concerted promotion efforts, the enrollment rate to CHF has remained low leaving the targeted population at risk of CHE [16, 20, 21]. To address this problem, the government reformed the CHF into the “improved Community Health Fund” (iCHF) in 2011, first as a pilot in Dodoma region. The reforms included a flat annual premium of about 15 USD covering 6 household members. The benefits package was also expanded to include x-rays, ultrasounds, in-patient services (excluding major surgery), and a referral system from District to Regional hospitals [22]. Table 1 summarizes the characteristics of the improved CHF (iCHF).

**Table 1:** Key characteristics of the improved CHF (iCHF)

S/N	Characteristics
1.	A reorganized structure that displays the different roles of the purchaser (CHF) and healthcare provider (health facilities)
2.	More advanced data management system including a central server with online and offline modes
3.	Active close‐to‐client strategy with village‐level enrollment officers
4.	Expanded range of services to include hospitalization and portability of CHF cards within the region (improved referral system)
5.	Active mobilization campaigns with social marketing strategies that involve both community‐based campaigns and mass media campaigns
6.	Each member of the household is given individual membership cards
7.	A flat rate premium in all districts equal to 30,000/ = per household that covers 6 household members

Source: Kalolo et al., 2018 [23]

### Literature review on CHE

The existing literature highlights a range of factors associated with CHE and the variation in the prevalence across countries. In Tanzania, three studies have assessed the incidence of CHE using the National Household Budget Surveys and they found that about 0.4% and 2.7% of the population experienced CHE at the 40% threshold of non-food expenditure (capacity to pay) [24–26]. Brinda et al. (2014), using data from the first round of the Tanzania National Panel Survey (TNPS) collected in 2008, found that 18% of the population experienced CHE at 40% threshold of non-food expenditure [7]. Macha (2015) found an incidence of 26.6% among 276 households when CHE was calculated based on the 10–20% threshold of the capacity to pay [10]. Studies from Mongolia, Malawi, Nigeria, and Vietnam found the incidence of CHE to be lower than 10% (i.e., 5.5%, 9.3%, 9.6%, and 9.9%, respectively) [27–30]. Studies conducted in Zambia, Kenya, and Uganda found incidences higher than 10% (i.e., 11.2%, 17.6%, and 23%, respectively) [31–33].

Previous studies on the determinants of CHE in various LMICs have primarily focused on demographic characteristics, disease patterns, and health-seeking behaviors. Some studies refer to higher age, higher educational level, sex of the household head, and occupation [10, 34–38], others refer to socioeconomic status and income [7, 8, 28, 34], while a few more mention chronic diseases and visits to health facilities [7, 34, 39].

A few studies have also explored the relationship between insurance status and CHE [34, 35, 39–41]. Two studies from China by Yang T. et al., (2016) and Li Y. et al., (2012) [34, 37], one study from Tanzania by Kihaule (2015) [38], and one multi-country study by Xu K. et al., (2003) [42], explored such relationships and found that being a member of a health insurance scheme reduced the incidence of CHE. Despite being insured, it is not uncommon for households to incur OOP expenditures, which may expose them to CHE [35, 36, 40]. A study by Aryeetey et al., (2016) from Ghana found that members of the National Health Insurance Scheme (NHIS) preferred to pay OOP so that they can get faster treatment [36]. Furthermore, informal fees, stock-outs of essential medicines at health facilities, and the exclusion of some services from the benefits package are also likely to expose patients to CHE [36].

Tanzania is currently considering implementing a mandatory health insurance scheme to raise additional funds for health [43]. Therefore, it is important to understand to what degree iCHF scheme contributes to better protection against CHE and how such protection varies across households belonging to different socioeconomic classes. Such knowledge may assist policymakers to improve the design of such schemes, which will ultimately enhance progress toward realizing the UHC goal. For this reason, this study aims to compare healthcare utilization and the incidence of CHE among improved Community Health Fund (iCHF) members and non-members in two rural districts located in central Tanzania.

## Methods

### Study design and setting

A cross-sectional study was used to collect primary data from Bahi and Chamwino Districts in Dodoma region between June to August 2019. Dodoma contains seven districts with a total population of nearly 2.3 million, of which 330,543 and 221,645 live in Chamwino and Bahi, respectively, according to the 2012 census [44]. The proportion of people enrolled in iCHF scheme in Dodoma region at the time of data collection was about 11%, however, there were some variations in coverage between the seven districts, with Bahi having a coverage of 16.5% and Chamwino of 17.4% [41].

### Sampling

A multistage sampling method was used to identify study participants. First, the two study districts were selected out of the seven districts in Dodoma. Second, four and five divisions were selected from Bahi and Chamwino, respectively. Third, for each division, two wards were selected, thus making a total of eight wards for Bahi and ten wards for Chamwino. Finally, 16 and 20 villages were selected from the wards in Bahi and Chamwino, respectively. The probability-proportional-to-size sampling approach was employed to obtain the sample size for each district by dividing the number of households in each district by the total number of households in the two districts multiplied by the estimated sample size (722), as explained in [45]. Out of the 722 households, 304 were from Bahi and 418 from Chamwino. Next, we used systematic random sampling by selecting every third household in each village to select the respondents. The office of the Village Executive Officer (VEO) in each village was selected as the central point. The trained research assistants walked in different directions (North, East, South, and West)approaching every third household.

### Data collection and variables

Six research assistants were trained for three days, followed by pretesting of the tools. Data were collected by these trained research assistants between June and August 2019. The questionnaire for this study was adapted from different sources [46–48]. The questions on health-related behavior, healthcare utilization, health expenditures, and insurance status were modified from the World Bank’s Living Standards Measurement Study questionnaire (LSMS) [49]. All respondents were interviewed face-to-face using a questionnaire with structured questions. After providing informed consent, the interviews started by asking the respondents whether or not they were members of the iCHF scheme. We did not interview households that were enrolled in other health insurance schemes.

The outcome variable was catastrophic health expenditure (CHE), which was defined as any health expenditure (HE) that exceeds 40% share of the total non-food expenditure [50, 51]. The main explanatory variables were iCHF enrollment status and socioeconomic status (SES). Enrollment status was measured as a binary variable with a “Yes” response if the respondent was a member of the iCHF scheme and a “No” if not a member. Socioeconomic status (SES) was measured as a categorical variable with 5 levels (lowest, low, average/middle, high, and highest)). Other explanatory variables are summarized in Table 2 and further details on how other variables concerning household expenditure (food, non-food, and health expenditure) were collected and measured are attached as Additional file 3.

**Table 2:** A list of the variables for the regression model

Variable	Variable labels
**Dependent variable**	
Catastrophic health expenditure (CHE)	1 = CHE > 40%, 0 = otherwise
**Main explanatory variables**	
Insurance status	1 = insured (iCHF member), 0 = noninsured (iCHF nonmember)
Socioeconomic status (SES)	1 = lowest,2 = low, 3 = average/middle, 4 = high, 5 = highest
**Healthcare and Health-related variables**	
Outpatient services (OPD)	1 = yes, 0 = no
Inpatient services (IPD),	1 = yes, 0 = no
Presence of chronic illness	1 = at least one household member with chronic illness, 0 = otherwise
Self-reported health state	1 = bad health, 2 = average, 3 = good health
**Socio-demographic variables**	
Age	1 = 18–25, 2 = 26–39, 3 = 40–59, 4 = 60 +
Sex	1 = male, 2 = female
Marital status	1 = unmarried, 2 = married
Household size	1 = 1–3, 2 = 4–6, 3 = 7–9, 4 = 10 +
Educational level	1 = No formal education, 2 = Primary education, 3 = Secondary education +
Number of children under 14 years	1 = 0, 2 = 1–4, 3 = 5–9 +

### Data analysis

To measure the socioeconomic inequality in the distribution of healthcare utilization among the iCHF members and non-members, we plotted the concentration curves and estimated the concentration index (CI) that ranges between -1 and 1. A positive value indicates a higher incidence among those in higher SES while a negative value would indicate a higher incidence among those in the lower SES [49]. To test whether the degree of inequality was statistically different, we conducted a dominance test. The dominance test is a common test for inequality measurement that uses the criterion that if one concentration curve (B) lies completely below the other concentration curve (A), then the inequality represented by curve A is higher than the inequality represented by curve B (curve A dominates curve B) [49, 52]. This type of test is done through a visual inspection of the concentration curves in comparison with the 45-degree line or another concentration curve. However, a visual inspection may not be sufficient to conclude whether or not dominance is statistically significant, therefore, the standard errors for the differences between the curves ordinates must be computed. Dominance will exist if the null hypothesis of non-dominance is rejected in favor of dominance when there is at least one significant difference between curves in one direction and no significant difference in the other i.e. *p* < 0.05 [49]. To calculate the CHE, OOP health expenditure was divided by non-food household expenditures and multiplied by 100 [7]

$$\mathrm{CHE}=\left(\frac{\mathrm{HE}}{\mathrm{NFE}}\right)*100$$

Where HE = average household monthly OOP health expenditure; NFE = average household monthly non-food expenditure. Thereafter, CHE was coded as ‘1’, if exceeded the threshold of 40%, and ‘0’ if otherwise. Multivariate logistic regression was employed to assess the associations between CHE and enrollment status and socioeconomic status (SES) when controlling for socio-demographic variables, health-related variables, and healthcare utilization variables. A list of the variables included in the regression model is available in Table 2. The results are reported as adjusted odds ratios and statistical significance was set at the 5% level. The statistical differences between groups were tested using the Chi-square statistical test and data analysis was carried out using STATA version 17 software.

## Results

### Socio-demographic characteristics of the households

Table 3 presents the socio-demographic characteristics of the sampled households compared across enrollment status (insured and non-insured) using a chi-square statistical test (p-value). The mean age of the household head was 44.67 years (18–90 years), 58% of the respondents were female and 73% were married. The majority of the household heads (72%) had completed primary education and 74% were farmers. The only variable that was significantly different across enrollment status was the presence of chronic diseases, which was more frequent among the insured. The mean household monthly income was $54 (2.2–870) and the average non-food expenditure (capacity to pay) was $44 (0.7–1,100).

**Table 3:** Socio-demographic characteristics of the households compared across enrollment status

**Variables**	**Enrollment status; n (%)**			
	**Insured**	**Noninsured**	**Total**	***p*-value**
**Age of the household head**				
*18–25*	13 (5.9)	29 (5.6)	42 (5.8)	0.147
*26–39*	63 (28.9)	176 (34.9)	239 (33.1)	
*40–59*	103 (47.3)	238 (47.2)	341 (47.2)	
*60* +	39 (17.9)	61 (12.1)	100 (13.9)	
**Sex of the household head**				
*Male*	84 (38.5))	220 (43.7)	304 (42.1)	0.201
*Female*	134 (61.5)	284 (56.3)	418 (57.9)	
**Marital status of the household head**				
*Married*	163 (74.8)	361 (71.6)	524 (72.6)	0.385
*Not married*	55 (25.2)	143 (28.4)	198 (27.4)	
**Education level of the household head**				
*No formal education*	36 (16.5)	91 (18.1)	127 (17.6)	0.350
*Primary education*	154 (70.6)	366 (72.6)	520 (72.0)	
*Secondary education and above*	28 (12.8)	47 (9.3)	75 (10.4)	
**Occupation of the household head**				
*Farmer*	160 (73.4)	375 (74.4)	535 (74.1)	0.776
*Non-farmer*	58 (26.6)	129 (25.6)	187 (25.9)	
**Household size**				
*1–3*	40 (18.4)	101 (20.0)	141 (19.5)	0.918
*4–6*	112 (51.7)	261 (51.8)	373 (51.7)	
*7–9*	56 (25.7)	122 (24.2)	178 (24.7)	
*10* +	10 (4.6)	20 (3.9)	30 (4.2)	
**Number of children under 14 years**				
*0*	29 (13.3)	72 (14.3)	101 (13.9)	0.496
*1–4*	151 (69.3)	357 (70.8)	508 (70.4)	
*5–9*	38 (17.4)	75 (14.9)	113 (15.7)	
**Chronic illness**				
*Yes*	91 (41.7)	164 (32.54)	255 (35.3)	0.018
*No*	127 (58.3)	340 (67.5)	467 (64.7)	
**Self-reported health state**				
Good	124 (56.9)	291 (57.7)	415 (57.5)	0.759
*Average*	74 (33.9)	175 (34.7)	249 (34.5)	
Bad	20 (9.2)	38 (7.5)	58 (8.0)	
	Mean (USD)	Minimum (USD)	Maximum (USD)	
Household monthly income	54	2.2	870	
Capacity to pay (non-food expenditure)	44	0.7	1,100	

### Healthcare utilization

From Table 4, it follows that 35.5% and 16.3% of the sampled households are reported to have utilized outpatient care (OPD) and inpatient care (IPD) respectively. More than half (50.9%) of those who utilized inpatient care, financed their medical expenses through the OOP payments modality followed by the group that used more than one means of financing modalities (26.3%). An example of a case with more than one payment modality (means of payment) would be the combination of premium payments and OOP payments.

**Table 4:** Healthcare utilization by enrollment status

	**Enrollment status; frequency (percentage)**			
**Variable**	**Insured**	**Noninsured**	**Total**	***P*-value**
**Outpatient services (OPD)**				
*Yes*	109 (50.0)	147(29.2)	256(35.5)	0.000
*No*	109 (50.0)	357(70.8)	466(64.5)	
**Inpatient services (IPD)**				
*Yes*	41 (18.8)	77 (15.3)	118 (16.3)	0.239
*No*	177 (81.2)	427 (84.7)	604 (83.7)	
**Type of health facility (OPD)**				
*Hospital*	8 (7.3)	15 (10.2)	23 (9)	0.137
*Health center*	56 (51.4)	66 (44.9)	122 (47.7)	
*Dispensary*	34 (31.2)	36 (24.5)	70 (27.3)	
*Clinic*	0 (0.0)	1 (0.7)	1 (0.4)	
*Pharmacy*	1 (0.9)	11 (7.5)	12 (4.7)	
*More than one*	10 (9.2)	18 (12.2)	28 (10.9)	
**Payment Modality (OPD)**				
*Out of Pocket (OOP)*	15 (13.8)	98 (66.7)	113 (44.1)	0.000
*Health Insurance*	55 (50.5)	0 (0)	55 (21.5)	
*Exemption*	9 (8.3)	31 (21.1)	40 (15.6)	
*More than one pay modality*	30 (27.5)	18 (12.3)	48 (18.8)	
**Type of health facility (IPD)**				
*Hospital*	14 (34.2	30 (38.9)	44 (37.3)	0.553
*Health center*	25 (61)	40 (52)	65 (55.1)	
*More than one*	2 (4.9)	7 (9.1)	9 (7.6)	
**Payment modality type (IPD)**				
*Out of Pocket (OOP)*	7 (17.1)	53 (68.8)	60 (50.9)	0.000
*Health Insurance*	13 (31.7)	0 (0)	13 (11.0)	
*Exemption*	4 (9.8)	10 (13.0)	14 (11.9)	
*More than one pay modality*	17 (41.5)	14 (18.2)	31 (26.3)	

When healthcare utilization was categorized by enrollment status and types of care sought, it follows from Table 4 that, 50% of the insured and 29.2% of the noninsured households had utilized outpatient care in the previous 4 weeks, while 18.8% of the insured and 15.3% of the noninsured households had utilized inpatient care in the previous 12 months. These findings confirm that the insured households had a higher healthcare utilization rate compared to the noninsured. The two groups (insured and non-insured) differ significantly in terms of OPD care utilization (*P* < 0.000) while there is no statistical difference in the utilization of IPD care (*P* < 0.239).

Furthermore, the proportion of insured households which utilized outpatient services and paid through OOP was 14% while 28% used more than one payment modality. This was not the case for the noninsured households where 66.7% and 12.3% of the households used OOP and the combination of different payment modalities, respectively. Concerning the inpatient care and the payment modality, we found that 17.1% and 41.5% of the insured households and 68.8% and 18.2% of the noninsured households incurred OOP expenditure alone or used more than one payment modality, respectively.

**Fig. 1:**
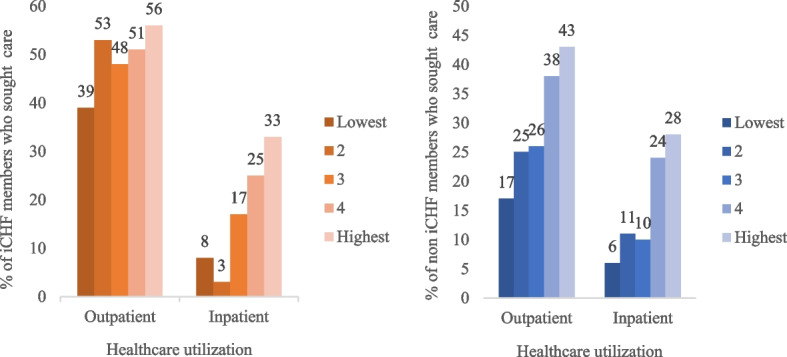
Proportion of households utilizing healthcare services by enrollment status

As shown in Fig. 1, we found that among the insured, the proportions of the households with the lowest and the highest SES that utilized OPD care were 39% and 56% respectively, while for the noninsured the proportions were 17% and 43%, respectively. This confirms that the households with low SES were less likely to utilize healthcare services compared to those with the highest SES, both for the insured and the noninsured. Overall, the insured utilized OPD and IPD care across all the wealth quintiles more than the noninsured; however, the utilization rate for those with low SES in the insured vs the noninsured had little impact on inpatient but potentially significant difference for outpatient care.

**Fig. 2:**
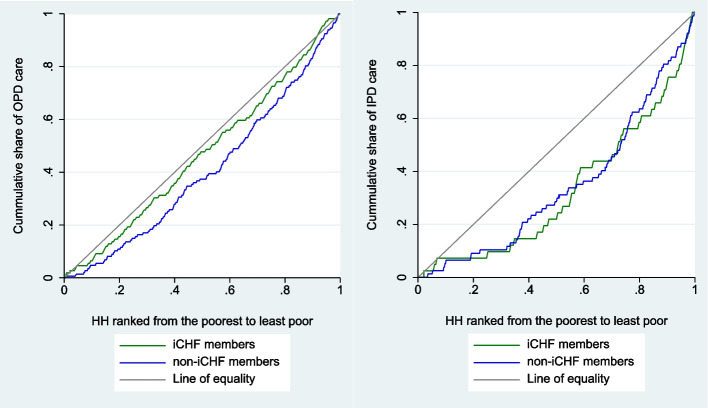
Concentration index curves for utilization of outpatient care (OPD) and inpatient care (IPD). Note: HH= household

Figure 2 presents the corresponding concentration curve of healthcare utilization among members and nonmembers. The figure indicates that utilization of OPD and IPD care was pro-rich among iCHF members and nonmembers. This means that the households with high SES had much higher utilization of OPD and IPD care compared to those with low SES regardless of the insurance status. However, utilization is more equitable for the insured relative to the non insured households in the case of OPD with concentration indices of 0.09 for the insured and 0.16 for the noninsured compared to the IPD care with CI of 0.38 for the insured and 0.29 for the noninsured.

The dominance test was statistically significant at *p* < 0.001 for both OPD and IPD care, suggesting that the noninsured strongly dominate the insured with respect to the utilization of healthcare services. From a visual inspection of Fig. 2, it follows that dominance exists in the utilization of OPD care among the noninsured because its curve lies above the insured curve and the two curves did not overlap one another. However, there was no dominance among the two groups (iCHF insured and noninsured households) in the utilization of IPD care since the concentration curves overlapped with one another. According to O’Donnell et al. (2007), dominance occurs only if one curve completely lies above the other [49].

### Catastrophic health expenditure

**Fig. 3:**
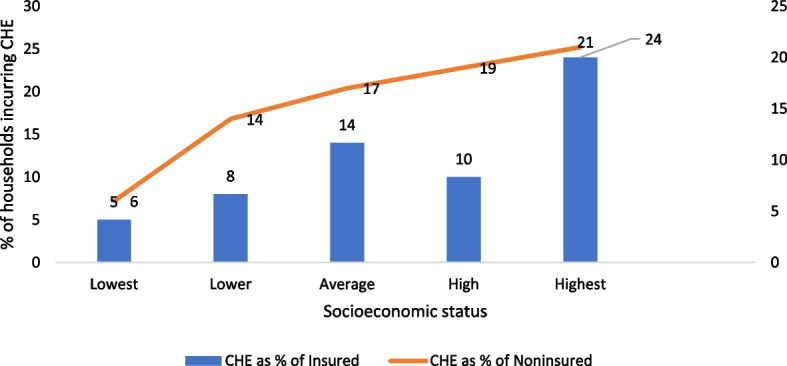
Proportion of households incurring CHE disintegrated by SES and enrollment status

The overall incidence of CHE was 15%; however, when disaggregated by enrollment status, the incidence was 15% among the noninsured and 13% among the insured. From Fig. 3, it is observed that regardless of enrollment status, the incidence of CHE increases with an increase in SES status (from the lowest to the highest SES). The only exception is for the insured when moving from the average/middle SES class to the high SES class.

### Determinants of catastrophic health expenditure

The regression results are presented in Table 5 and the model output is attached as Additional file 2. The results show that the insured households were less likely to incur CHE compared to the non-insured households. The odds of the insured household incurring CHE were 0.41 times less compared to the non-insured (OR = 0.41, 95%CI: 0.27–0.63) when controlling for the other factors that were likely to influence CHE. Moreover, household heads reporting a good health state, having secondary education or more, and who were married, were less likely to incur CHE compared to their counterparts. For example, the odds of a household head with secondary education or more incurring CHE was 0.07 times less compared to the household head without no formal education (OR = 0.07, 95%CI: 0.01–1.02).

**Table 5:** Multivariable logistic regression analysis of the determinants of CHE

Variables	OR (95% CI)	*P*-value
**iCHF enrollment status**		
*Non-member*	1	
*Member*	0.41 (0.27–0.63)	0.000***
**Socioeconomic status**		
*Lowest*	1	
*low*	2.45 (1.14–5.27)	0.022**
*Average/Middle*	4.05 (3.71–4.42)	0.000***
*High*	1.22 (0.67–2.24)	0.514
*Highestt*	2.43 (2.12–2.80)	0.000***
**Outpatient services (OPD)**		
*No*	1	
*Yes*	9.18 (4.66–18.10)	0.000***
**Inpatient services (IPD)**		
*No*	1	
*Yes*	37.69 (36.53–38.88)	0.000***
**Reported health state**		
*Bad*	1	
*Average*	0.83 (0.76–0.92)	0.000***
*good*	0.67 (0.39–1.12)	0.127
**Presence of chronic illness**		
*No*	1	
*Yes*	1.49 (1.34–1.68)	0.000***
**Age of the household head**		
*18–25*	1	
*26–39*	0.66 (0.35–1.27)	0.215
*40–59*	0.73 (0.50–1.06)	0.099*
*60* +	0.52 (0.34–0.81)	0.003***
**Sex**		
*Male*	1	
*Female*	0.91 (0.67–1.23)	0.543
**Education level**		
*No formal education*	1	
*Primary education*	0.85 (0.44–1.68)	0.648
*Secondary education and above*	0.07 (0.01–1.02)	0.052*
**Marital status**		
*Unmarried*	1	
*Married*	0.41 (0.31–0.54)	0.000***
**Household size**		
*1–3*	1	
*4–6*	1.48 (0.25–8.72)	0.664
*7–9*	1.01 (0.09–11.75)	0.996
*10* +	1.73 (0.81–3.69)	0.156
**Number of children under 14 years**		
0	*1*	
*1–4*	1.77 (0.39–8.00)	0.458
*5–9* +	1.23 (0.08–18.53)	0.883

Notes: ^***^, ^**^ and ^*^ denote 1%, 5% and 10% significance level (*p*-value), respectively

For the socioeconomic status (SES), households with at least one member with chronic illness, and households with at least one member that had received IPD care, or OPD care, were more likely to experience CHE. SES was positively associated with CHE, however the odds ratio first increased from the lowest to the average/middle), then decreased when moving to high and, again increased when moving to the highest SES. Households that belonged to the low, average/middle, and the highest SES were 2.45, 4.05, and 2.43 times more likely to incur CHE compared to those belonging to the lowest SES. Not surprisingly, the odds ratios for OPD and IPD are very high. Households that received inpatient care were 37.69 times higher likely to incur CHE compared to their counterfactuals (OR = 37.69, 95%CI: 36.53–38.88) while for those who received outpatient services the odds ratio was 9.18 times higher relatively to those that did not (OR = 9.18, 95%: 4.66–18.10).

## Discussion

This paper compared healthcare utilization and the incidence of catastrophic health expenditure (CHE) among households enrolled into the improved Community Health Fund (iCHF) and those not enrolled. This topic is of considerable interest given the ongoing Tanzanian efforts to reach Universal Health Insurance coverage. The incidences of CHE provide us with insights about the ability of a health system to provide risk financial protection for its citizens as well as the financial burdens that are carried by households.

Our findings show that the insured households utilized healthcare services (both outpatient and inpatient) to a higher degree than the noninsured households. One of the advantages of voluntary health insurance is to provide financial risk protection and improve healthcare accessibility [36, 53–55]. Our findings show that the iCHF scheme has managed to improve access to care among the members than non-members. The observed improvement is likely to follow from healthcare being less costly, however, a higher degree of utilization may also, at least in part, be explained by adverse selection. According to David et al., (1998), individuals who expect high future healthcare costs would prefer to be insured [56]. Since the iCHF scheme in question does not screen its potential clients before purchasing the premium, and since the potential clients know more about their health conditions than others, then adverse selection may arise in the sense that the utilization rate among the insured becomes higher relatively to the noninsured. Our findings are in line with findings from Ghana in the sense that those insured by the Ghanaian National Health Insurance Scheme (NHIS) were more likely to seek formal healthcare compared to noninsured [55].

A second observation is that households in the highest SES class utilized both outpatient and inpatient services more frequently than those in the lowest SES class and were also more likely to incur CHE. These findings are in line with studies conducted in Nigeria and Mongolia [28, 57, 58]. A recent study using 26.3 million claims data from the National Health Insurance Fund (NHIF) in Tanzania, showed that the lowest-income group had a lower probability to visit accredited facilities than the reference middle-income category [53]. A possible explanation for such findings could be that households with higher SES in contrast to those with lower SES are able and willing to pay for healthcare services [59]. Several studies have pointed out that high OOP payments discourage households with low SES from seeking appropriate healthcare services, and instead opt to go to pharmacies/drug shops or traditional providers [35, 38, 60, 61].

In this study, we found that overall, 15% of the households experienced CHE at a 40% threshold of the capacity to pay (non-food expenditure). This incidence is smaller compared with the 26.6%, which was reported by Macha (2015) but quite similar to 18% reported by Brinda et al., (2014), both in Tanzania [7, 10]. fThe incidence of CHE estimated from our study seems to be higher compared to other studies from Tanzania [24–26]. A study by Mchenga et al., (2017) found that about 1% of the population experienced CHE at a 40% threshold of the capacity to pay, while WHO (2016) and Binyaruka (2020) found that, at 40% threshold, about 0.4% and 2.7% of the population in Tanzania suffered CHE [24, 25, 29]. Possible explanations could be that these studies used relatively old data from Household Budget Surveys (HBS) while our data was collected more recently and from districts that are susceptible to CHE. Compared to studies done in other countries, our estimates are relatively similar to those reported in Malawi (9.3%), Nigeria (9.6%), Zambia (11.2%), Kenya (17.6%), and Uganda (23%) [27, 29, 31–33, 62]. It should be noted that the above studies differ in terms of study settings and health system context.

Our results show that the incidence of CHE was higher among the noninsured households than the insured. This is not surprising, since health insurance per definition provides financial risk protection. However, quite a high share of insured households were also confronted with CHE. We can only speculate that these households purchased healthcare services that were not included in the iCHF benefit package or because medicines were out-of-stock forcing them to purchase from private pharmacies and drug shops. Furthermore, treatments for some common Non-Communicable Diseases (NCD) are not covered by Ichf scheme, meaning that OOP remains the only option to finance such expenditures. Our findings are similar to the findings of other studies which also found that CHE was more pronounced among the noninsured households compared to the insured households [28, 62, 63].

The study found that CHE was influenced by socio-economic variables, healthcare variables, and health-related variables. For the socioeconomic variables, CHE was associated with age (60 + groups), education (secondary education and above), marital status (married), and SES. For the healthcare variables, CHE was associated with a household having at least one member who received inpatient care in the last 12 months or outpatient care in the last month. For the health-related variables, CHE was associated with households having at least one member suffering from chronic diseases and a household head that report having a good health status.

A negative relationship was observed between the age of the household head and CHE. This suggests that, as the age of the household head increases, the likelihood of experiencing CHE decreases. A possible explanation for this could be the exemption policy that matters for the elderly, which excuses them from paying OOP at public health facilities. Similar findings were reported in a previous study that identified an inverse relationship between higher age and CHE [9]. However, studies from Uganda, India, and China found that households with older household heads were more likely to face CHE compared with households having younger household heads [37, 51, 64].

Our results have also revealed that a higher educational level (secondary level and above) and being married were negatively associated with CHE. A study conducted in China found that the incidences of CHE decreased with a higher educational level [34]. The explanation could be that educated people are more forward-looking (time preferences) implying that future outcomes are given more weight relative to less educated people. Our finding concerning marital status contradicts Choi et al., (2016) who found that household heads who were married or living together had higher odds of incurring CHE than those who were divorced or separated [65]. One possible reason for our finding can be that single-headed households typically are more vulnerable (marginalized), in terms of household income and the number of dependants per adult, thus making it more difficult to avoid CHE.

The results show that SES typically has a positive association with CHE, although the odds were not consistent across all classes. This provides a clear picture that the average household is more vulnerable to CHE due to a combination of income and spending where those with low SES are less likely to access care, unlike the ones with high SES who are more likely to access care because they can afford it. Another possible explanation could be that as SES increases, so does the household capacity to pay for health care, which may translate to more OOP payment without exposing them to CHE compared to those with low SES whose budgets are more constrained and hence becomes difficult to visit health facilities when sick. Our findings are in line with other studies, which also found that low SES increased the probability of households incurring CHE [7, 10, 28, 64, 66].

Self-reported health status and households having at least one member with chronic diseases were found to be associated with CHE, same as households having at least one member who sought IPD or OPD care. These findings are in line with what has been reported by other studies [7, 10, 34, 39]. Healthcare needs are probably key determinants of CHE and our findings are as expected since a low health state and the presence of chronic diseases may imply a low household income (due to low productivity) in combination with a high demand for healthcare services that include services that are not covered by the benefit package in question.

### Strengths and limitations of the study

This study was faced with some limitations, we, therefore, request caution with the interpretation of its findings. First, this was a cross-sectional study conducted in two districts in one region, which limits the generalization of the results beyond the study districts. Secondly, the health expenditure data reported by the study participants may have been misrepresented due to recall bias. Respondents were asked to state the quantity of resources purchased or the expenditure on food, non-food items and health services in the past 4 weeks, or the past 12 months. We feel that it might have been difficult for the respondent to accurately remember the value and quantities of some consumed items. Another reason for underestimation is that we only took into consideration those who had visited the health facilities within the last month for OPD care or last year for IPD care. If the respondent had not visited the health facility, then the expenditure was not captured. Despite these limitations, our findings are robust in the sense that they are comparable to previous studies that used the same methodology. Furthermore, household expenditures rather than household income is in the literature considered to be the most reliable measure of wealth status for study settings like ours because people in the informal sector often have no formal or reported income sources, which might result in measurement error [67–70].

## Conclusion

The study found that the utilization of healthcare services was relatively higher and the incidence of CHE was lower among households enrolled in the iCHF insurance scheme compared to those not enrolled into the scheme. Despite the odds of an insured household incurring CHE being lower compared to noninsured households, we found that being insured did not eliminate the possibility of experiencing CHE. Therefore, more studies are needed to establish the reasons behind the relatively high incidence of CHE among insured households. Our findings also show that healthcare utilization and incidence of CHE were lower among households with low SES compared to those with higher SES. Therefore, researchers and policymakers must seek to identify other possible barriers beyond enrollment into health insurance that hinder the utilization of healthcare services among households with low SES when formulating policies for Universal Health Coverage in Tanzania.

### Supplementary Information

**Additional file 1.** Proposed household questionnaire on insurance status, health status, access to healthcare, expenditures, socioeconomic status, and demographic characteristics.

**Additional file 2. **Model output for Multivariate Logistic regression.

**Additional file 3.**Data collection and variable measure.

## Data Availability

The datasets used and/or analyzed during the current study are available from the corresponding author on reasonable request.

## Abbreviations

CHF: Community Health Fund
iCHF: Improved Community Health Fund
CBHIs: Community –Based Health Insurance Schemes
CHE: Catastrophic Health Expenditure
CI: Concentration index
GDP: Gross Domestic Product
IPD: Inpatient services
LMICs: Lower-Middle-Income Countries
OOP: Out-of-Pocket
OPD: Outpatient services
Tshs: Tanzanian shillings
USD: United State Dollar
WHO: World Health Organization
