# Supplementary material for: Healthcare utilization and catastrophic health expenditure in rural Tanzania: does voluntary health insurance matter?
Source: BMC Public Health. 2023 Aug 17;23:1567. doi: 10.1186/s12889-023-16509-7 (PMC10436390; doi:10.1186/s12889-023-16509-7)
Supplement: Supplementary file 1 — Additional file 1. Proposed household questionnaire on insurance status, health status, access to healthcare, expenditures, socioeconomic status, and demographic characteristics. [file 12889_2023_16509_MOESM1_ESM.docx]

**Questionnaire–English versions**

**Proposed household questionnaire on insurance status, health status, access to healthcare, expenditures, socioeconomic status, and demographic characteristics.**

**SECTION A: SOCIO-DEMOGRAPHIC AND ECONOMIC INFORMATION OF THE RESPONDENTS (Household head or representative of the household head)**

1. ID………………….

| 02  Age (in years)  18-23.…1  24-29.….2  30-35…...3  36-41…...4  42-47…...5  48-53…...6  54-59…...7  60+……...8 | 03  Sex  Male…...…1  Female.….2 | 04  Marital status  Single …………...1  Married.................2  Living together….3  Divorced...............4  Widowed..............5  Separated………..6 | 05  How many people, including yourself, live in this household?  …………….. | 06  How many members of your household are less than 14 years of age?  ……………. | 07  Your highest level of education being completed  No formal education………….1  Primary school ……2  Ordinary level Secondary school …3  Advance level secondary school.…4  Vocational training..5  College/ ………......6  University…………7 | 08  Your religion  Christian...........1  Muslim..............2  Traditional…….3  None believer….4  Other (specify)...5 | 09  Your Main Occupation?  Farmer…………….1  Livestock breeder…2  Retired …………....3 Craftsman……...….4 Merchant………….5  Civil Servant………6  Daily wage laborer……………..7  Not earning any income…………….8  Other (specify)…………...9 | 010.  What is the average monthly income of your household?  (*This should be the sum of what all household members bring in taken together)*  …………...Tshs |
| --- | --- | --- | --- | --- | --- | --- | --- | --- |

**SECTION B: FOOD EXPENDITURES**

I am now going to ask you some questions about food consumption expenditures.

| 011. Have your household purchased any food items in the last seven days (1 week)?  Yes……………...1  No………………2 (go to 013) | 012. How much did your household then use in total to purchase food items in the last seven days (1 week)?  ...................Tshs. | 013. Have your household consumed any food items that were produced at your home in the last one week?  Yes…………….1  No……………...2 (go to 015) |
| --- | --- | --- |
| 014. If you were to buy the same amount of food from the shop or market, how much would you have to pay in total?  ....................Tshs. | 015. Have your household, during the last seven days /1 week) consumed any food items that were given to your household by relatives, friends, neighbours etc.  Yes………….1  No……………2 (go to 017) | 016. If your household were to buy the same amount from the shop or market, how much would you have to pay in total? ........................ |

**SECTION C: NON-FOOD EXPENDITURES**

I am now going to ask you questions about non-food expenditures

| 017.  In the last ONE MONTH (the last 30 days), how much has your household approximately spent on the following items? | | | | 018.  In the last ONE YEAR (the last 12 months), how much has your household approximately spent on the following items? | | | |
| --- | --- | --- | --- | --- | --- | --- | --- |
| Items | Qnty (Kgs/Lts | | Amount (Tshs) | Items | Qnty(Kgs/Lts) | Amount spent (Tshs) | |
| Personal care items (tooth paste, lotion, soap, etc. ) ………………………………………………...1  Electricity……………………………..………2  Petrol and/ diesel and/kerosene.……...………3  Charcoal and firewood……………………….4  Batteries …………………………… …5  Salary permanent worker (Maid, cattle herder) ……………………………………………….6  Salary seasonal worker (daily laborers) ……7  Public transport………………………….…8  Air time……………………………………9  Shoes and clothes………………………..10  Household appliances and utensils……...11  Others (specify)………………………….12 |  |  | | Education (school fees, stationary, etc.) …………………………………..1  Fertilizers or seeds………………2  Building materials……………….3  Agricultural equipment………….4  Business inputs………………......5  Radio andTV……………………...6  Sewing machine…………………...7  Cell phone………………………….8  Bicycle……………………………...9  Furniture (chairs, beds, tables etc) ...10  Household repairs………………….11  Others (specify)……………………12 |  | |  |

**SECTION D: HEALTH EXPENDITURES**

I will now ask some questions on health care expenditures

| 019.  During the last ONE MONTH (last 4 weeks), have you, or any other member of your household, demanded outpatient care?  Yes………1  No ………2 (jump to 024) | 020.  At what type of institution were the outpatient services demanded?  *(you can tick more than one response)*  Hospital……………...1  Health centre…………2  Dispensary……………3  Clinic…………………4  Pharmacy/Drug shop…5  Traditional Healer……6  Other (Specify)………7 | 021.  Was the institution visited a public or a private facility?  *(you can tick more than one response)*  Public…………....1  Mission………......2  Private…………....3  Designated……….4 | 022.  How were the received services paid for? *(You can tick more than one response)*  Out of Pocket.......1  Health Insurance...2  Exemption.............3  Other(Specify)......4 |
| --- | --- | --- | --- |
| 023.  If you made out of pocket payments, what was the total amount (in Tshs) being paid by the household during the visit during the the last month (last 4 weeks)?  Registration fee Tshs…………………………………1  Consultation fee Tshs…………………………………2  Tests and supplies (including gloves) Tshs. ..................3  Drugs Tshs…………………………………………….4  Unofficial payments Tshs.........……………………….5  Transport costs (to and from the facility) Tshs….……6  Other (specify)………………………………..………7 | 024.  During the last ONE YEAR (last 12 months), has any member of your household been admitted to stay overnight at a healthcare facility (in-patient services)?  Yes……1  No………2 (jump to 028) | 025.  At what type of healthcare facility was the household member admitted?  *(you can tick more than one response)*  Hospital……………...1  Health Centre…………2  Other (Specify)………...3 | 026.  How did you pay for the in-patient services?  *(You can tick more than one response)*  Out of Pocket.......1  Health Insurance...2  Exemption.............3  Other(Specify)......4 |
| 027.  In total, how much did your household spend on the following items in connection with the healthcare facility admission?  Hospital Bills Tshs..………………………..….…1  Drugs Tshs..…………………………………....…2  Supplies (syringes, gloves, etc.) Tshs…………….3  Unofficial payments Tshs..……………………….4  Transport costs (to and from the facility) Ths… ...5  Other (specify)………………………………...…6 |  |  |  |

**SECTION E: ASSESSMENT OF THE HEALTH STATUS OF THE RESPONDENT**

| 028.  In general, how would you assess your average health situation in the last 30 days?  Very good……1  Good…............2  Fair………......3  Bad……….….4  Very bad…….5 | 029.  Do you or any of your household members have a permanent illness (a chronic disease)?  Yes...…1  No……2 | 030.  Do you worry that yourself, or any of the household members, will get a future health problem?  Yes………….1  No…………...2 |
| --- | --- | --- |

**SECTION F: MEMBERSHIP STATUS**

| 031.  Are you enrolled into the iCHF scheme?  Yes……….1  No………..2 (go to 038) | 032.  How many household members are registered members of the iCHF scheme?  .............number | 033.  For how long time have you/your household been members of the iCHF scheme?  …….Months/Years | 034.  Are you/your household planning to stay enrolled into the iCHF scheme in the future?  Yes……1  No…….2 |
| --- | --- | --- | --- |
| 035.NON-members  Have you/your household been former members of the CHF/iCHF scheme?  Yes………1(go to 040)  No……….2 | 036. NON-members  Do you/your household consider to become a future member of the iCHF scheme?  Yes….1  No.….2 | 037.  Are there any members of your household that are members of any other health insurance scheme besides iCHF?  Yes……1  No…….2 (go to 042) | 038.  What type of insurance scheme?  NHIF………………………...1  PHI…………………...…...…2  SHIB…………………...…....3 |

**Thank you for your time and participation**
