# Supplementary material for: Healthcare utilization and catastrophic health expenditure in rural Tanzania: does voluntary health insurance matter?
Source: BMC Public Health. 2023 Aug 17;23:1567. doi: 10.1186/s12889-023-16509-7 (PMC10436390; doi:10.1186/s12889-023-16509-7)
Supplement: Supplementary file 2 — Additional file 2. Model output for Multivariate Logistic regression. [file 12889_2023_16509_MOESM2_ESM.docx]

Model output for Multivariate Logistic regression

| Number of obs |  | 722 |
| --- | --- | --- |
| LR chi2(23) |  | 238.11 |
| Prob > chi2 |  | 0 |
| Pseudo R2 |  | 0.3976 |
| Log likelihood |  | -180.35696 |
