# Supplementary material for: Healthcare utilization and catastrophic health expenditure in rural Tanzania: does voluntary health insurance matter?
Source: BMC Public Health. 2023 Aug 17;23:1567. doi: 10.1186/s12889-023-16509-7 (PMC10436390; doi:10.1186/s12889-023-16509-7)
Supplement: Supplementary file 3 — Additional file 3.Data collection and variable measure. [file 12889_2023_16509_MOESM3_ESM.docx]

**Data collection and variable measure**

The questionnaire consisted of three categories of household expenditures, which were food expenditure (FE), non-food expenditure (NFE) and healthcare expenditure (HE). The healthcare utilization variables were measured as binary outcomes with yes/no responses. The respondents were first asked whether any of the household member had visited a health facility for either (a) outpatient services (OPD) within the last 4 weeks or (b) inpatient services (IPD) within the last year. Thereafter, they were asked to report health expenditures of the household for outpatient and inpatient care for the relevant recall periods. These expenses included registration fees, consultation fees, laboratory diagnosis, treatment costs, food, transport and accommodation costs, both for the patients and their companions.

Furthermore, the respondents were asked about the average monthly household expenditure on food and non-food items. This approach of collecting data on household expenditure for food, non-food and health is a standard method introduced by the WHO and the World Bank and has been used in various studies that measure living standards [1,2]. The expenditure data were collected in the local currency (Tshs) and then converted to USD using the prevailing exchange rate of 1 USD = 2,318 Tshs on 23^rd^ June 2021. The expenditure on items with a recall period of one year were divided by 12 to convert them into the same recall period of 1 month.

We used total household consumption expenditure as a measure of socioeconomic status/wealth (SES), hence households were ranked into five wealth quintiles (socioeconomic groups) based on their aggregate consumption expenditure [3]. SES was first constructed as an index of the total household expenditure by ranking households from the lowest to the highest household. The SES index was then divided into equal fifth (20%) quintiles. The first quintile represents the lowest 1/5 of values from 0-20% followed by the second quintile from 20-40%, to the fifth quintile including the highest 1/5 from 80-100%.

**Reference**

1. Grosh M, Glewwe P. Designing household survey questionnaires for developing countries: lessons from 15 years of the living standards measurement study. Vol. 1, The World Bank. Washington, D.C.: The World Bank; 2000. 356 p.

2. Ssewanyana S, Kasirye I. Estimating Catastrophic Health Expenditures from Household Surveys: Evidence from Living Standard Measurement Surveys (LSMS)-Integrated Surveys on Agriculture (ISA) from Sub-Saharan Africa. Appl Health Econ Health Policy. 2020;18(6):781–8.

3. Deaton A, Zaidi S. Guidelines for constructing consumption aggregates for welfare analysis. World Bank Living Stand Meas Study Work Pap. 2002;135:64–5.
